# Supplementary material for: High risk injection drug use and uptake of HIV prevention and treatment services among people who inject drugs in KwaZulu-Natal, South Africa
Source: PLoS One. 2023 May 12;18(5):e0281030. doi: 10.1371/journal.pone.0281030 (PMC10180682; doi:10.1371/journal.pone.0281030)
Supplement: Attachment — Submitted filename: PONE-D-23-01161.docx [file pone.0281030.s001.docx]

Review

PONE-D-23-01161

This is a mixed-methods study (n=45) to understand current drug use practices and access to HIV prevention and treatment services for PWID in KwaZuluNatal, South Africa. The authors used respondent-driven sampling to identify a population at high risk for HIV/viral hepatitis and disconnection from HIV services. The eligible population was individuals who reported injecting opiates within the past 6 months from Durban, KwaZulu-Natal, South Africa. They found high rates of practices that increase HIV/viral hepatitis risk. The author include local unsafe injection practices such as “flashblooding” or “bluetoothing” that could threaten the gains made in HIV prevention and treatment. Of the 35% of participants living with HIV in the sample, only 40% accessed antiretroviral therapy within the past year. There was no prior hepatitis C testing by self report. The importance of this study is access to a high risk population for HIV/hepatitis C, which has been underreported in the literature and has low utilization of HIV and hepatitis C prevention and treatment services. The paper would benefit from a greater emphasis on opportunities for novel program innovation to improve access of HIV/hepatitis prevention and treatment services. I have provided suggestions under major comments for potential avenues for innovation. Please also address how other resource limited countries address high risk injection practices that could be applied to KZN. Overall the paper is well-written and by addressing comments below, should merit publication.

Major comments

Is there any further published literature on bluetoothing / flashblooding or other similar practices in other countries and how the authors’ study findings compare? Please add this to the discussion to set the context of the significance of findings.

The paper could benefit from emphasizing opportunities for high impact novel interventions, in paragraph 2 of the Discussion. For example, the sample noted high rates of homeless and high rates of overdosing. Please address the need and feasibility to address, housing, food security, and Narcan availability, which may improve survival and adherence to harm reduction and HIV/hep C treatment programs? How can HIV/hep C programs be adapted to find this population that has high rates of homelessness.

Furthermore, how do the findings necessitate more locally tailored risk assessments to accurately identify high risk local practices. Are injection drug use risk screening tools adequate to account for practices that include bluetoothing? For example, Dawn K. Smith, Jeffrey H. Herbst et al. doi: 10.1097/ADM.0000000000000123, albeit a U.S. based sample, does not account for local variations in practice. How would more direct solicitation of these practices in one on one interactions reduce stigma and allow consumers of harm reduction services to be more forthcoming of their use?

Please also address how other resource limited countries are addressing high risk injection practices that could be applied to KZN.

Minor comments

Line 24—briefly define endocarditis for the non clinical audience

Results Lines 71 and 72 are a duplication from the methods. Please move the dates of interviews up to the methods Lines 39-41 where the authors report the same information and delete this sentence from the results.

Discussion Line 112-6 -rewrite for better clarity

In addition to the sentence on Line 116, “Nationally, needle exchange programs, harm reduction services, and 117 medication assisted treatment are often difficult to access either due to their high-priced, fee-for118 service models or limited availability. (24)” Can you mention the restrictions on providers to medication assisted treatment /buprenorphine (e.g. are there special restrictions, licenses,/fees required for health care providers to deliver that restricts its implementation?).
